# Comparative study of the environmental footprints of marinas on European Islands

**DOI:** 10.1038/s41598-021-88896-z

**Published:** 2021-04-30

**Authors:** Noelia Cruz-Pérez, Jesica Rodríguez-Martín, Celso García, Florin Ioras, Nicholas Christofides, Marco Vieira, Manfredi Bruccoleri, Juan C. Santamarta

**Affiliations:** 1 Department of Agricultural, Nautical, Civil and Maritime Engineering, Universidad de La Laguna, Santa Cruz de Tenerife, Spain; 2 Technical Department and Projects in Engineering and Architecture, Universidad de La Laguna, Santa Cruz de Tenerife, Spain; 3 Departament of Geography, University of the Balearic Islands, Palma, Spain; 4 Buckinghamshire New University, Queen Alexandra Road, Wycombe, UK; 5 Frederick University, Nicosia, Cyprus; 6 ACIF-CCIM - Associação Comercial e Industrial Do Funchal - Câmara de Comercio e Industria da Madeira PT, Funchal, Portugal; 7 University of Palermo, Palermo, Italy

**Keywords:** Climate-change ecology, Environmental economics, Freshwater ecology, Urban ecology, Environmental social sciences

## Abstract

Ports have been key elements in Europe's economic development. This situation is even more relevant on islands, which are highly dependent on the maritime sector. Consequently, over the years, ports with diverse functionalities have been established both in mainland Europe and on its outlying islands. This article discusses the environmental impact of leisure marinas on European islands, especially as they are closely linked to economic development through tourism. The aim is to study the environmental impact of these infrastructures by determining the carbon and water footprints of marinas on European islands in the Atlantic and the Mediterranean. The results obtained enable the authors to make recommendations in order to reduce the overall environmental footprint of marinas on islands, considering that these territories are much more vulnerable to climate change than mainland locations in Europe.

## Introduction

Historically, the European Union has always had an important connection with the sea, as its trade relations with the rest of the world have relied heavily on its seaports^1^. This maritime dependence is still evident. European Directive 2019/883 states that *“… The Union’s maritime policy aims to ensure a high level of safety and environmental protection”*^2^. If at first, consideration was given only to the development of a maritime sector and port infrastructures focused on trade, over the years, the concept has evolved and new knowledge acquired and, nowadays, ports are devoted to a range of purposes^3^. In recent years, ports have been developed for tourism related activities, and cruise ships and maritime passenger transport vessels dock in areas built specifically for them^4^. There has also been a growing number of leisure ports or marinas built for boats with mainly recreational or leisure purposes^5^.

In this article, the authors focus on leisure marinas, as they now constitute their own segment within the maritime sector due to their number and characteristics^6^. Up till now, marinas have been studied far less than commercial ports, and they are often just included in a certain area of a larger commercial port^7^. However, their relevance is growing. Indeed, the recreational boating sector generates a positive economic impact on the places where marinas are established^8^. Yet, despite being a driving force for local job creation, their existence may also be associated with maritime pollution in their area of operation^9^. Such emissions from ships in ports have an effect on climate change, but also affect the health of people living in coastal areas^10^. Increasing numbers of researchers, governments and international organizations have been considering the impacts of leisure marinas on the environment in light of the rapid development of the global tourism industry and the burgeoning environmental issues of climate change and water resource scarcity^11^. Consistent with this focus, many marinas footprint analyses have emerged in recent years, including ecological footprint analysis^12^, tourism carbon footprint analysis^13^, and tourism water footprint analysis^14^, which share the research target of better integrating tourism industry development with the protection of the ecological environment^15^.

Sport marinas have become the main base for nautical tourism, which is increasingly growing in Europe^16^. Tourism is a sector that has been growing steadily over the years, and different models have been created to exploit it^17^. One of them is the one related to the sea, where sport marinas bring together those people who make stopovers with their private boats when they are doing leisure trips^18^, as well as activities related to the sea such as excursions to see cetaceans, recreational activities (paddle surfing, jet skis, etc.), which enjoy a notorious importance in the tourism that takes place in the European islands^19^. Increasingly, the marinas are also hosting restaurants and stores of various kinds, which attract tourists also for leisure activities on land^20^. The activity related to nautical tourism has not stopped growing in Europe, especially in countries with a great nautical tradition such as Spain, Italy and France^21^. In fact, Italy has the second highest number of pleasure boats per capita in Europe, and the production of pleasure boats in this country represents an income of approximately 2.9 billion euros^22^.

The port operations that have the greatest impact on the environment are those conducted at diesel fuel dispensing stations, and the repairs and maintenance of ships in dry dock. The products handled in these operations such as petrol, fuel and its derivatives, wastewater, detergents, paints, glue, resin, protectors and used oils all have negative effects on the marine environment. Port dredging activities also cause significant changes in the physical and chemical conditions of the environments^8^. Other actions related to boating sector with impacts on the environment are the losses suffered by ships during navigation, the management of solid waste^23^, the discharge of waste oil or bilge water and alteration of the seabed by anchoring or mooring and the movement of the propellers. The impact depends on the number of ships on each route^24^ and the vessel size, whether it is motor or sail, and the number of crew. It also depends on the mode of operation. For example, pressure from leisure marinas differs from that of freight ports because the latter have associated logistics and industrial services that are not needed in marinas^25,26^.

The purpose of this paper is to contribute to the body of knowledge by using case studies to assess carbon and water footprint in the context of environmental impacts of leisure marinas and by considering shortcomings, proposes supply chain as areas for further developing the environmental footprint.

## Analytical framework

The calculation of carbon footprints can be addressed by following two basic methodological approaches^27^. The first is the business-oriented method, which consists of collecting data on the direct and indirect consumption of materials and energy by an organization and translating it into equivalent CO_2_ emissions in order to have an inventory of emissions. The Green House Gas Protocol, developed by the Word Resources Institute and the Word Business Council for Sustainable Development, is the most widely used guide by companies, both large and small, to establish an inventory of their GHG emissions and thus calculate their carbon footprint^28^. The importance of this protocol is that it has been the basis for many other methods and initiatives. The ISO 14,064: 2006 standard (parts 1 and 3) is a second tool which follows the company approach^29^. Unlike the Green House Gas Protocol, the ISO standard is an international standard verification guide for companies to prepare and report on their greenhouse gas inventory. In contrast to these approaches, there is another product-focused methodology. Product-focused tools collect the material and energy consumption at each stage of a product's life until it is placed on the market. And, once all the information is available, it is translated into terms of CO_2_ emissions. Finally, the composite accounting method or MC3 is a mixed approach, oriented to both the organization and the product^30^. Unlike the previous methods, the information in the composite method is obtained from the organization's accounts.

**Figure 1:**
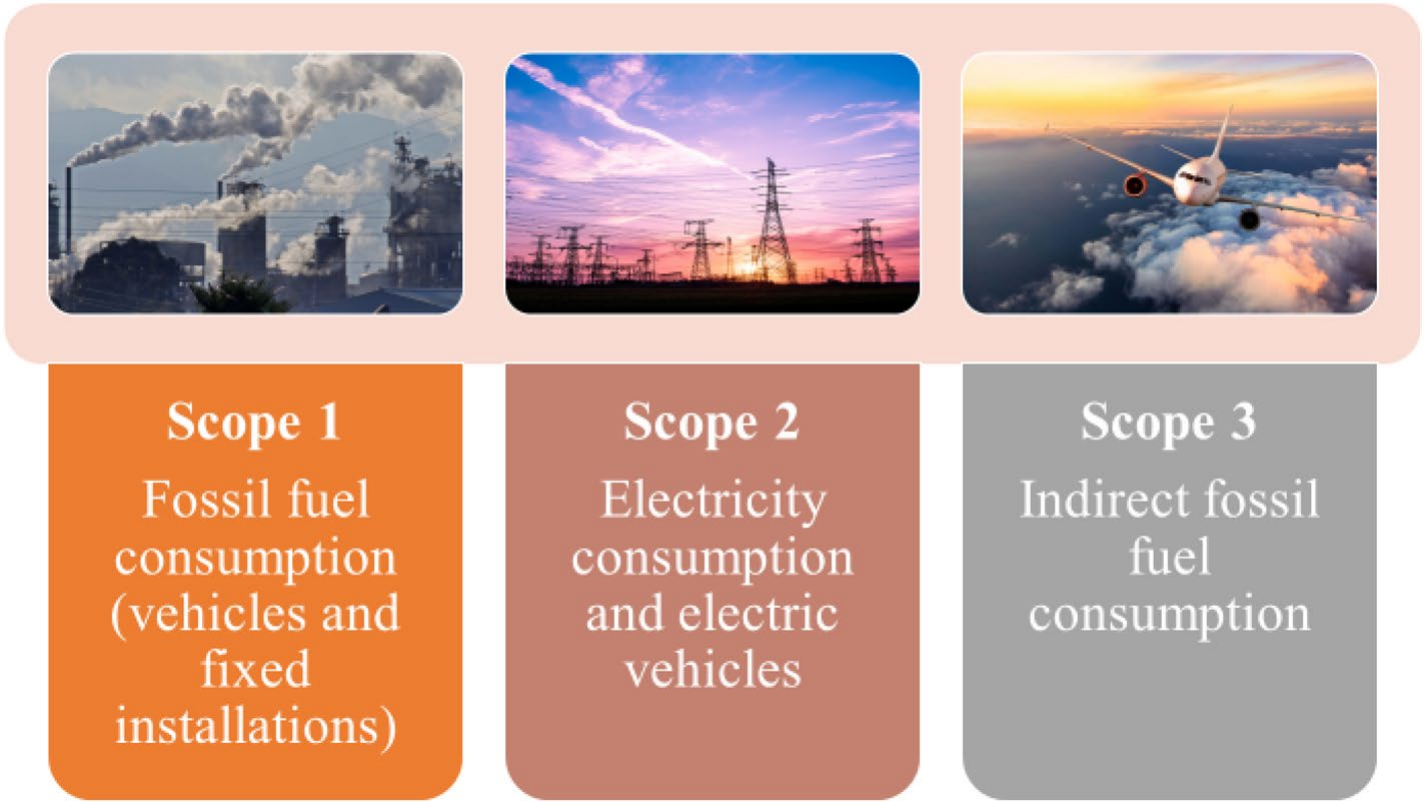
The three scopes of the carbon footprint. Prepared by authors.

GHG emissions can be classified into three types (Fig. 1). Direct emissions or so-called Scope 1 emissions are those that come from the fuels that the organisation uses in its processes or in transport. Indirect emissions or Scope 2 emissions are those related to the generation of electricity acquired by the organisation^31^. Third, there are the so called other indirect emissions or Scope 3 emissions that include indirect emissions of any type and electricity. Finally, if the register includes the carbon footprint of capital goods, works and all fixed assets, the methodology used is complete.

If we compare leisure marinas with freight ports, we observe differences in the way they operate and, therefore, in the emissions they generate. Freight ports also have associated logistics and industrial services that increase their carbon footprint considerably^26^. Another aspect that influences an increase in GHGs in freight ports as opposed to leisure ports, is the way in which ships obtain energy while they are berthed^25^. Traditionally, cargo ships use generators while in the dock, which triggers the emission of greenhouse gases under Scope 1.

The water footprint accounts for the use of drinking water required by an activity for its proper development, as well as the study of water pollution^32^. The water footprint is composed of three components: blue, grey and green water footprint. Green water corresponds to water from precipitation, which is not lost through runoff and is incorporated into the soil or vegetation^33^. Blue water, on the other hand, corresponds to the fraction of the hydrological cycle that is transformed into surface or subway runoff and is consumed by incorporation or evaporation in the evaluated process. It feeds the flow of rivers and aquifer reserves, and can also be captured artificially through the construction of reservoirs^34^. Finally, grey water is a theoretical concept that refers to the pollution of the resource. It represents the volume of water needed to reduce the load of pollutants to meet current water quality standards^35^. Regarding water footprint, the main contaminants found in the waters of leisure marinas are: heavy metals, traces of antifouling paints^36^, pesticides, suspended solids, etc.^37^. One other important factor in leisure marina management and which is relevant for this study is the direct water consumption by the marina, which also provides an indication of the potential volume of water contaminated by activity in a leisure marina.

The total direct water consumption is estimated by calculating the blue water footprint, green water footprint and grey water footprint. In the case of sports marinas, the direct water footprint has been estimated considering only blue water (drinking water obtained from a supply source), excluding the volume of green and grey water. The reasons why the volumes of green and grey water have been discarded are as follows: green water accounts for the volume of rainwater that is incorporated into a product (this aspect being particularly important when agricultural products are studied, but becoming irrelevant in the rest of the cases)^38^; grey water takes into account the volume of water that would theoretically dilute the pollutants generated as a result of the process to which the blue water has been subjected to concentrations lower than its maximum admissible concentration according to the most restrictive legislation in force^39^.

Islands are particularly vulnerable to climate and environmental changes^40,41^. Climate observations, which began in the mid-nineteenth century, provide a global view of the observed variability and changes in the planet's climate. According to the Intergovernmental Panel on Climate Change (IPCC)^42^, global average surface temperature has been increasing steadily since the late nineteenth century, and each of the last three decades has been warmer than any other on record, with the 2000s being the warmest decade on record^43^. Therefore, the rise in sea level (which may compromise the existence of existing port facilities), as well as the increase in temperatures^44^ and changes in rainfall patterns^45^, are three factors that directly affect territories such as the European islands^46^. It is therefore necessary to study the environmental impact of the activities carried out on the islands, with particular importance being given to tourism^47^ and agricultural activities^48^. Only by establishing the current emissions of each activity or product can improvement plans and ecological transition policies be established^49^. To this end, two internationally recognized environmental indicators are the carbon footprint and the water footprint, which make it possible to measure the emissions and pollution caused by a company or activity.

## Methods

### Case study selection and characterization

**Figure 2:**
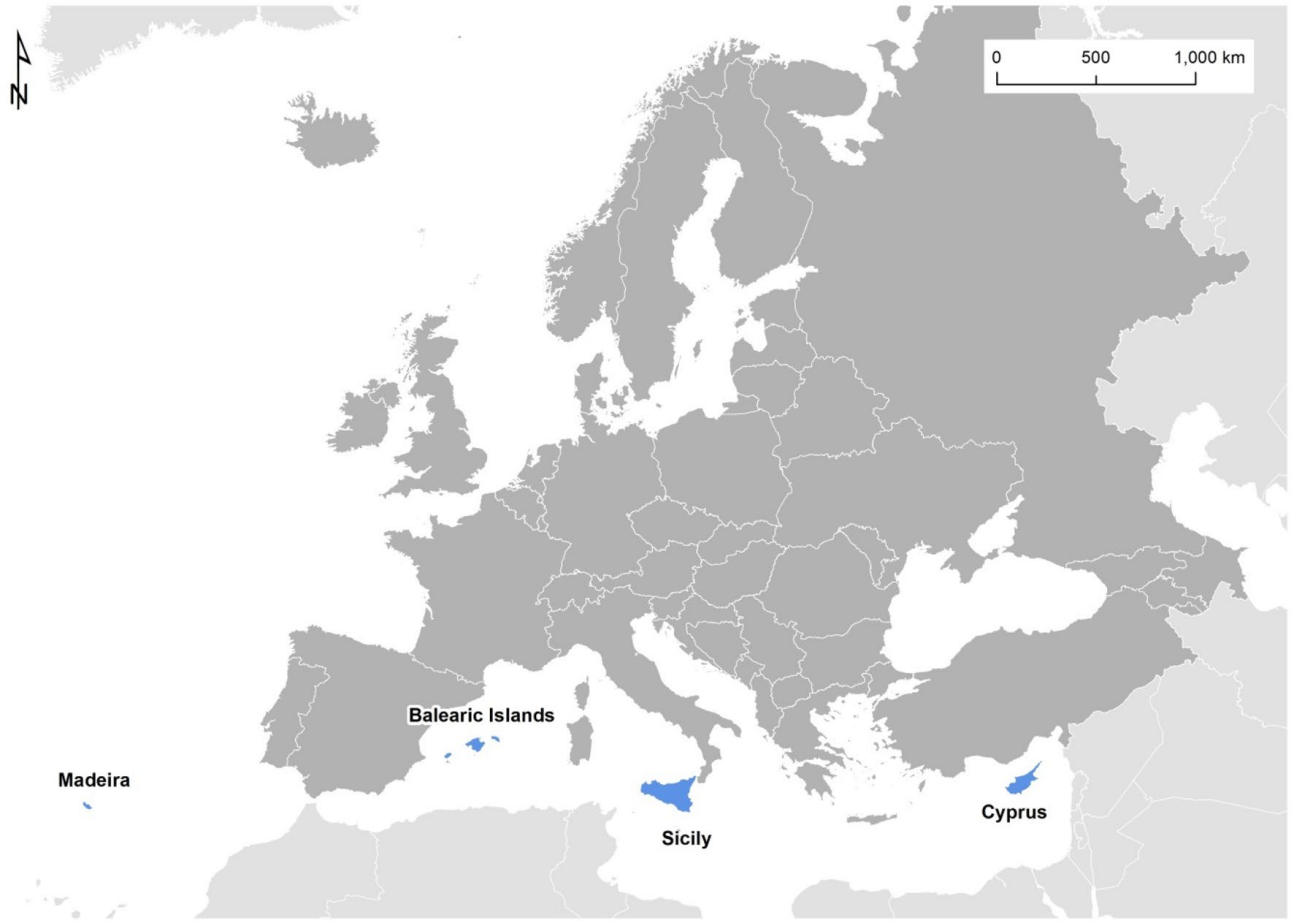
Location of the marinas analysed. *Source*: Prepared by the authors and generated with ArcMap version 10.4.1.

In this study, we have selected leisure marinas located on European islands, since on these islands, they have proliferated along the coast due to demand from tourism and local inhabitants^50^. The study includes two marinas located in Madeira (Portugal), one in Cyprus, seven in the Balearic Islands (Spain) and two in Sicily (Italy) (Fig. 2). The objective has been to identify the carbon and water footprints of these marinas for the year 2019 and to identify differences in operational management among them. In this study, the authors sought to analyse the environmental impact of European marinas from the point of view of carbon footprint and water footprint. Current known studies related to marinas are more focused on water pollutants derived from the operations conducted in the port^51,52^, the study of emerging pollutants derived from sunscreens (among others)^53^, waste management^54^, as well as the modification of the existing marine biology in these areas^55,56^. Nevertheless, from a complete environmental point of view, there is still no similar study that studies greenhouse gas emissions and implicit the carbon footprint from European marinas.

### Data collection and analysis

For the purpose of this study, in order to obtain data and be able to calculate the carbon footprint and water footprint, a survey was conducted aided by a web-based questionnaire sent out by email to those directly in charge of the marina. The questionnaire was intended to reveal the way in which the marinas are operated, as well as the main characteristics of each one (Table 1). All experimental protocols were approved by University of La Laguna (Tenerife, Spain). Besides, our study was approved by Bucks New University, Research Ethics Panel Oct 2019. Moreover, in order to conduct this enquiry, informed consent was obtained from all subjects.

**Table 1:** Questionnaire sent to the selected marinas. *Source*: prepared by the authors.

Question	General information	Unit	Indicator	Scope
**Questionnaire**				
Q1.1	Type of port	Transit/Base	–	3
Q1.2	Number of workers	nº	–	3
Q1.3	Average daily commute of workers to the marina	km	Carbon	3
Q1.4	Average daily commute of tourists to the marina	km	Carbon	3
Q2	Number of berths	nº	–	–
Q3	Vessel dimensions	m × m	–	–
Q4	Activities most frequently carried out by ships	Open-ended question	Carbon/Water	–
Q5.1	Separate waste collection	Yes/No	–	3
Q5.2	Frequency waste collection	Times/Year	Carbon	3
**Maintenance activities**				
Q6	Developed by who	Open-ended question	–	3
Q7	Frequency of these tasks	Times/Year	Carbon	3
Q8	Hot water production system	Open-ended question	Carbon	2*
**Consumptions**				
Q9	Electric	kWh	Carbon	2
Q10	Diesel fuel	liters	Carbon	1
Q11	Water	m^3^	Water	–
**Suppliers**				
Q12	Number of suppliers	nº	Carbon	3
Q13	Frequency of visits	Times/Year	Carbon	3
Q14	Type of vehicle	Open-ended question	Carbon	3
**Office**				
Q15	Number of people working	nº	Carbon	3
**Restaurants**				
Q16	Bar, Cafeteria, Restaurant, etc	Type	Carbon/Water	3
Q17	Quantity	nº	–	3
Q18	Energy source	Type	Carbon	2*
**Visitors**				
Q19.1	Quantity	nº	Carbon	3
Q19.2	Vehicle	Type	Carbon	3

*If the power source is the electrical grid. However, if the energy source were a diesel-powered generator set, this section would go to Scope 1.

The methodology used for the calculation of the carbon footprint is based on the GHG Protocol system. Such methodology enables the calculation of an organisation's carbon footprint in accordance with relevant guidelines and regulations. This system accounts for a port's emissions considering the three Scopes. Scope 1 includes emissions related to diesel. Diesel fuel is used mainly in generators and in vehicles owned by the marina. Scope 2 includes everything related to electricity, whether it is the electricity consumption of the marina (where it is important to know whether the origin of this electricity is from renewable sources or not)^57,58^ or electric vehicles that the marina company may own. Scope 3 is the most general of all, and includes all aspects considered relevant in the generation of emissions due to the services provided to the marina^59^: for example, diesel fuel from the suppliers' vehicles on their way to the marina where they are going to deliver their goods, tourists who come there to board a boat that goes on a whale watching trips, etc. Once each of the emissions has been identified with its corresponding Scope, these units (kWh, litres, etc.) must be converted into tonnes of equivalent CO_2_, using the corresponding emission factors available through official sources.

The quality and completeness of the data requires a systematic procedure for the collection of information. Following this premise, a web-based survey has been developed where most of the questions are open-ended and cardinal in nature. There are only three multiple choice questions. Question 10, which refers to the use of fuel by the marina, corresponds to scope 1; questions 8, 9 and 18, linked to the total electricity consumption of the marina, in scope 2, and questions 1, 5, 6, 7, 12, 13, 14, 15, 16, 17 and 19, which allow to approximate the fuel consumed by the vehicles of visitors, suppliers and waste manager, in scope 3. Questions 4 and 11 are related to the total water consumption of the marina or the water consumption used in maintenance activities. The rest are questions aimed at formulating recommendations to reduce and/or offset the footprints. The survey is addressed to the marina manager who should also support his answers with invoices or other documents.

The scope of study of marinas encompasses the total area of the port, i.e. both the water area where the boats dock and the land area where different services such as offices, repair shops, restaurants, stores, toilets, waste collection point, parking, facilities, etc. are housed.

Emission sources associated with fixed operations (those located in the shore area) include facilities for administration, maintenance, cleaning and showering activities, restaurants, stores and hotels. Whatever the case may be, the number of personnel that marina has, the source of energy to carry out the activities and the consumption of water and electricity used on average when these tasks take place are quantified.

Mobile sources include vehicles used by marina personnel, visitors, suppliers and waste managers. In all cases, the number of workers and the average round trip distance in kilometres per working day per employee between their usual residence and the marina have been considered. The same applies to suppliers, tourists and waste managers, considering in each case the nearest tourist area or industrial estate, where applicable.

The water footprint has been calculated using the Water Footprint Network (WFN) approach, which differentiates between direct water footprint and indirect water footprint. The direct footprint is the water consumption of the marina throughout the year, which is used for the gross calculation, and the indirect footprint is the water consumption of the products consumed by the marina. This last indirect element has been discarded, as marinas offer services and not products, so only the direct water consumption of each marina consumed in m^3^ has been considered.

As explained in the Methods section, for the calculation of the direct water footprint of the marinas, the green and grey components have been eliminated, considering only the blue water. The blue water associated with a service is estimated from the consumption per type of service and the number of users per service. In the case of sports marinas, blue water consumption was obtained from the water bills of the marina and outside companies that provide some type of service in the marina.

## Results

In total, 12 European marinas have been studied: two from Madeira (Portugal), two from Sicily (Italy), one from Cyprus and seven from the Balearic Islands (Spain). The results of the carbon and water footprints are presented in Table 2.

**Table 2:** Results of the carbon and water footprints of the 12 European marinas analysed.

	Units	Madeira1	Madeira2	Cyprus	Sicily1	Sicily2	Mallorca1
		Value	Value	Value	Value	Value	Value
Total Scope 1	t CO_2_ eq	0	523,5	4,257,2	0	0	16,2
Total Scope 2	t CO_2_ eq	5,9	50,5	1,063,7	4,7	6,2	3,362
Total Scope 3	t CO_2_ eq	57,2	113,1	616,3	151,8	19,8	485,8
Carbon footprint	t CO_2_ eq	63,1	687,1	5,937,2	156,5	25,9	3,864,1
Water footprint	m^3^	2,720	0	10,668	1,500	500	77,000

		Mallorca2	Mallorca3	Mallorca4	Mallorca5	Menorca1	Menorca2
Total Scope 1	t CO_2_ eq	0	10,2	3	0,2	0	3,7
Total Scope 2	t CO_2_ eq	19,0	4,426	796,3	278,6	89,9	34,7
Total Scope 3	t CO_2_ eq	147,5	863,6	356,9	71,5	150,2	349,5
Carbon footprint	t CO_2_ eq	166,5	1,316,3	1,156,1	350,2	240,1	388
Water footprint	m^3^	17,576,3	4,183	12,723	4,356,0	8,065	1,207

Within Scope 1, only emissions corresponding to fixed installations have been accounted for, since no marina has responded that it owns vehicles. In Scope 2, the emissions corresponding to the electricity used by the marina for its daily activity have been counted. In Scope 3, the emissions corresponding to the gasoline of the vehicles of suppliers, workers, tourists and waste managers, in their relationship with the marina (i.e., trips to and from the port, with the corresponding frequency in each case), were included.

Moreover, to better understand the results obtained, the main characteristics of each marina studied are presented in Table 3.

**Table 3:** Main characteristics of the 12 European marinas studied.

	Madeira1	Madeira2	Cyprus	Mallorca1	Mallorca2	Mallorca3
No of boats	210	337	630	488	40	745
Diesel consumption (L)	0	210,000	1,707,655	6,500	0	4,072
Electricty consumption (kWh)	14,368	123,156	2,594,414	8,200,000	46,296,4	1,079,598
No of suppliers	2	2	115	125	5	200
No of workers	12	9	31	36	9	90

	Mallorca4	Mallorca5	Menorca1	Menorca2	Sicily1	Sicily2
No of boats	200	70	650	155	80	30
Diesel consumption (L)	1,188,3	76,5	0	1,491	0	0
Electricty consumption (kWh)	1,942,103,7	679,512	219,377	84,742,3	11,506	15,000
No of suppliers	70	30	5	72	130	40
No of workers	15	5	10	7	7	2

There are four marinas with a carbon footprint of over 1000 t of equivalent CO_2_. Three of them are in Mallorca and one in Cyprus (Cyprus has the largest carbon footprint of all). By relating the data in Table 2 with the data in Table 3, we can see that these marinas are the ones with the greatest capacity for mooring boats. However, one of the marinas with the lowest carbon footprint also has a high number of moorings (Menorca 1), but the main difference is that Menorca marina 1 does not have diesel consumption, which prevents Scope 1 from skyrocketing and so it is in the second group of marinas, which are those with consumption between 100 and 1,000 t of equivalent CO_2_.

## Discussion

Marinas are located in coastal areas, which sometimes place them close to tourist areas. Despite this, they are activities that have developed independently, which has led them to lag behind other tourism activities in terms of sustainability^60^. The environmental aspect of ports has been studied from several perspectives, mainly how the gases emitted by the port activity affect the inhabitants of coastal cities^61^, the presence of chemicals in coastal waters^62^, the presence of microplastics^63^, etc. However, in this study we have focused on the environmental impact that the activity and the facility have on the environment, using the indicators of carbon footprint and water footprint.

The assessment of the carbon footprint is only mandatory for two scopes: 1 and 2. Nevertheless, when studying companies that provide a service, as in the case of marinas, it is highly recommended to calculate scope 3 because it provides interesting information about the operations related to our activity and their impact on the environment. This is because the existence of a port causes a large amount of road travel associated with it, thus increasing emissions within Scope 3.

The transport of goods in commercial ports has been the subject of numerous environmental studies due to its importance^64–66^. In this study we have found that in marinas their impact is also notable, since in most cases scope 3 is higher than scope 1 and/or 2.

It should be noted that marinas’ activities depend entirely on external suppliers and companies to provide an adequate service to their customers. This boosts the economy of the area in which they are located, but also significantly increases CO_2_ emissions from vehicles that come daily to the marinas to facilitate their day to day activity^67^, hence the need to convert marinas in places where circular economy concepts are introduced^68^. One of the measures that could solve this circumstance, would be the use of electric vehicles, something that we have observed that it is increasingly taking place but a long way from where it should be to achieve desire result in the locations studied^69^. Therefore, the number of workers and number of suppliers directly impact on Scope 3 of the carbon footprint, making the marinas with the largest footprint the ones with the most suppliers and workers (due to the average daily trips considered with their own vehicles).

There are only two marinas with a carbon footprint below 100 t of equivalent CO_2_, one located in Sicily and the other one is in Madeira. Both have several similarities, such as, they do not use fossil fuels (there is no Scope 1), and electricity consumption is quite low. Therefore, the consumption of fuel/oil directly by the marina is one of the aspects that clearly marks the amount of emissions into the atmosphere, meaning that, if the port's dependence on fossil fuels is reduced and electricity supply is entirely from renewable energies, an elimination of scopes 1 and 2 is achieved by the port^70^. In other words, the port would be able to eliminate the greenhouse emissions generated directly by this activity^71^.

In the marinas of Cyprus and Mallorca 1, 2, 3 and 4, high electricity consumption is observed, quite related to a large number of moorings in the port. With regard to electricity, the ecological transition within the electricity sector is one of the key aspects of the European Union's Energy-Climate Package^72^. Indeed, the EU has set itself the objective of reducing the continent's emissions related to electricity production by 27% by 2030^73^. A stronger focus on renewable energies would naturally offset a large part of Scope 2 emissions and significantly reduce greenhouse gas emissions from activities. In Spain, the electricity sector accounts for up to 65% of the country's total emissions^74^, which is why the country has been working on the development of wind and solar energy for more than a decade now. Wind energy in Spain currently accounts for 52% of renewable energy production^75^. The case of Cyprus presents an even greater challenge, as its electricity system is totally isolated as an island and shows little flexibility when it comes to introducing renewable energies into it^76^.

Hence, water withdrawal seems not too high in any of the studied marinas, especially when compared to other water intensive activities on the islands such as hotels^77^, agriculture^78^ and urban consumption^79^. However, the water consumption in some of them, for example the 77,000 m^3^ from marina Mallorca 1, is the same amount of water consumed by the municipality of Mancor on the island of Mallorca, with 1500 inhabitants, during 2019. Similarly, the total volume of 116,000 m^3^ used by the five marinas in Mallorca equals the water consumed in 2019 for the municipality of Petra, with 2,800 inhabitants^80^; or the volume charged by the cruise ships during April–October 2016 in the harbour of Palma^81^. The water footprint for marinas makes it possible, for the first time, to evaluate their water consumption in a context, especially in the Mediterranean islands, where water resources are limited, and droughts have a strong environmental and socio-economic impact. Current climate scenarios predict freshwater availability to be problematic in the Mediterranean islands^82^.

It should be considered that green and grey water have not been considered, and that we have limited ourselves to studying the consumption of drinking water for this activity. Therefore, the analysis of seawater pollutants in the port is not included in the study, since all the ports studied pump their wastewater outside the port facilities, thus following waste management regulations. Furthermore, there is a correlation between energy consumption and water consumption. Those marinas generating high water footprints are also those marinas that have a larger carbon footprint. This may be due, among other things, to the source of energy used to heat the shower/toilet water in the marinas.

## Conclusions

Marinas are revitalizing activities for the area where they are located, boosting the economy and the tourist offer of the area. At the same time, since they are facilities that provide a service and do not manufacture a product, they depend to a great extent on outside suppliers to carry out their activity. This means that the scope 3 is high in all cases, since suppliers, tourists, workers, visitors, etc. travel to the marinas on a daily basis. Therefore, it is considered interesting as a future line of research, to conduct a study to minimize these trips and involve electric vehicles in suppliers, in the rental vehicles of tourists and in the residents of the marinas.

In a large number of cases, Scope 1 is already minimal or non-existent, implying that direct dependence on fossil fuels appears to be on a downward trend within European navies. Therefore, if within Scope 2 a service is contracted that comes from renewable energies, we would have the two main scopes that depend directly on the marina compensated. This means that if marinas eliminate the use of fossil fuels and the energy sources they use are renewable, they would not have a direct carbon footprint, only an indirect one.

In any case, nautical tourism is a growing trend on the European continent, so it is important to seek the sustainability of these sites, which are large consumers of electricity and require a large number of external services for their operation.

In the case of water footprint, consumption is, individually, lower than other activities on the islands. However, the total water uses by marinas on each island represents an important amount in the context of water scarcity in the Mediterranean islands. Therefore, every single effort to reduce water consumption by the marinas will be welcomed, especially under the present and future consequences of climate change impact on fresh water availability.
